# Circ_nuclear factor I X (circNfix) attenuates pressure overload-induced cardiac hypertrophy via regulating miR-145-5p/ATF3 axis

**DOI:** 10.1080/21655979.2021.1960462

**Published:** 2021-09-01

**Authors:** Jun Pan, Zhenjun Xu, Guanjun Guo, Can Xu, Zhizhao Song, Kunsheng Li, Kai Zhong, Dongjin Wang

**Affiliations:** Department of Thoracic and Cardiovascular Surgery, The Affiliated Drum Tower Hospital of Nanjing University Medical School, Nanjing, Jiangsu, China

**Keywords:** Hypertrophy, circRNA, miRNA, ATF3, Nfix, circNfix

## Abstract

Cardiac hypertrophy can cause heart failure. However, the mechanisms underlying the progression of cardiac hypertrophy remain unclear. Emerging evidence suggests that circular RNAs (circRNAs) play a critical role in cardiac hypertrophy. However, the association between circ_nuclear factor I X (circNfix) and cardiac hypertrophy remain largely unknown. Therefore, the aim of the present study was to explore the role of circNfix in cardiac hypertrophy. In order to detect the function of circNfix in cardiac hypertrophy, cardiomyocytes were stimulated with angiotensin II (Ang II) to mimic the pathogenesis of the disease. In addition, pressure overload-induced cardiac hypertrophy in a mouse model was established using transverse aortic constriction (TAC) surgery. The mechanism via which circNfix regulated cardiac hypertrophy was investigated using RNA pull-down and luciferase reporter assays, and fluorescence *in situ* hybridization (FISH). circNfix was downregulated in Ang II-treated cardiomyocytes. Similarly, circNfix expression was markedly downregulated in mice following TAC surgery. In addition, circNfix overexpression significantly prevented the progression of cardiac hypertrophy in TAC-treated mice. Luciferase activity and RNA pull-down assays indicated that circNfix could indirectly target activating transcription factor 3 (ATF3) by binding with microRNA (miR)-145-5p in cardiomyocytes. miR-145-5p overexpression or ATF3 knockdown could reverse the effects of circNfix in Ang II-treated mouse cardiomyocytes. circNfix attenuated pressure overload-induced cardiac hypertrophy by regulating the miR-145-5p/ATF3 axis. Therefore, circNfix may serve as a molecular target for cardiac hypertrophy treatment.

## GRAPHICAL ABSTRACT

**Figure:**
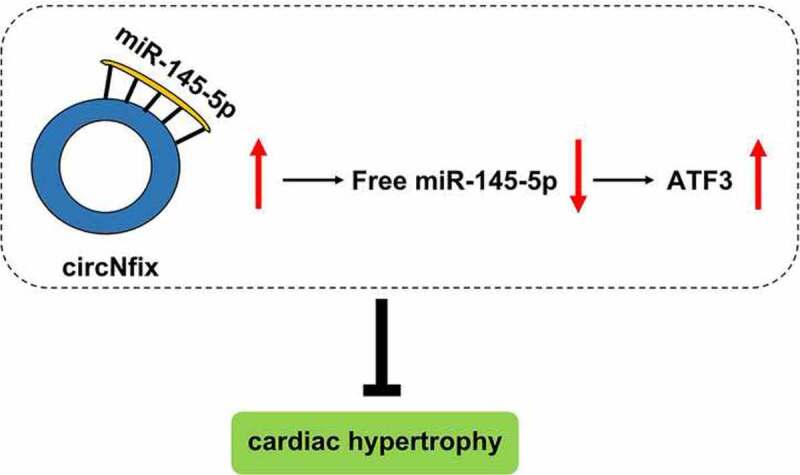

## Introduction

Cardiac hypertrophy is an adaptive response to various forms of cardiac dysfunction [1–3]. Cardiac hypertrophy is characterized by an increased cardiomyocyte size and protein synthesis [1,4]. In addition, cardiac hypertrophy is an important risk factor for heart failure, cardiac sudden death, myocardial infarction and arrhythmia [5–7]. During physiological myocardial hypertrophy, normal cardiac function is maintained, and an increased pumping ability is sometimes observed [8,9]. However, pathological myocardial hypertrophy is characterized by cardiac dysfunction and can result in systolic dysfunction and heart failure [10]. Evidences have shown that pathological myocardial hypertrophy can be induced by cytokines, growth factors, hypertension, obesity or myocardial infarction [11–13]. Recently, RNA-based therapy has been shown to be a promising new treatment strategy for cardiovascular diseases, including cardiac hypertrophy [14–16].

Circular RNAs (circRNAs) are produced by back-splicing and have no 5ʹ to 3ʹ polyadenylated tails [17–20]. Recently, circRNAs have been found to be abundant and highly stable [21]. Several studies have indicated that circRNAs are involved in several physiological processes and play a key role in various diseases [22,23]. They also serve a vital role in the initiation and progression of cardiovascular disease [22,24,25]. For instance, upregulated circ_ASXL transcriptional regulator 1, circ_nuclear factor I X (circNfix), and circ_calmodulin regulated spectrin associated protein 1 have been found to be involved in cardiac maturation [26], and heart-related circRNA (HRCR) has been proven to induce cardiac hypertrophy [27]. In addition, circNfix could play a vital role in the development of cardiovascular disease; circNfix silencing could inhibit the cardiac injury induced by myocardial infarction through sponging microRNA (miR)-214 and regulating Y-box binding protein 1 expression [28].

However, the expression and role of circNfix in cardiac hypertrophy remains unclear. Thus, in this study, we aimed to investigate the association between circNfix and cardiac hypertrophy. In the present study, a mouse and a cell model of cardiac hypertrophy were used to explore the role of circNfix during the progression of cardiac hypertrophy. We found that circNfix could attenuate Ang II–induced cardiac hypertrophy *in vitro* and TAC-induced cardiac hypertrophy *in vivo* by regulating the miR-145-5p/activating transcription factor 3 (ATF3) axis.

## Materials and methods

### In vivo *model of pressure overload-induced cardiac hypertrophy*

C57BL/6 mice (age, 7–9 weeks) were obtained from Beijing Vital River Laboratory Animal Technology Co., Ltd. Following anesthesia with 1% sodium pentobarbital (intraperitoneal injections; 50 mg/kg), the mice were treated with TAC surgery, as described previously [29]. Briefly, the mouse aorta was ligated using a silk suture (6–0, 26 G blunt needle) to yield a 0.46-mm diameter constriction of the aorta. Mice in the sham group underwent the same surgery without ligation. The study was approved by the Committee of The First Affiliated Hospital of Nanjing Medical University. All procedures were in line with the National Institutes of Health Guide for the Use and Care of Laboratory Animals.

The mice were classified into three groups (n = 8 per group), including the sham, TAC and TAC + circNfix groups. The adenovirus overexpressing circNfix (ad-circNfix; 1 × 10^8^ pfu; HANBIO, Shanghai, China) was injected into the myocardium of the left ventricle 7 days prior to TAC surgery. The heart weight to tibia length ratio (HW/TL ratio) was recorded after the surgery.

### Cell culture and transfection

Neonatal C57 mice (day 3) were obtained from Beijing Vital River Laboratory Animal Technology Co., Ltd. Mice were sacrificed with CO_2_ overdose (30% volume/min) and tissues were sectioned into 1–3 mm^3^ pieces. Neonatal mouse cardiomyocytes (NMCMs) were isolated using 0.1% collagenase II and trypsin (Beyotime Institute of Biotechnology). 293 T cells were obtained from the American Type Culture Collection (ATCC). NMCMs and 293 T cells were cultured in Dulbecco’s Modified Eagle’s medium (DMEM; Thermo Fisher Scientific, Waltham, MA, USA) containing 10% fetal bovine serum (FBS, Thermo Fisher Scientific) and maintained at 37°C with 5% CO_2,_ as described previously [29].

ad-circNfix was synthesized by HANBIO (Shanghai, China). NMCMs were cultured in DMEM containing 12% FBS at 37°C with 5% CO_2_ overnight. After that, the adenovirus was then added to the plates for 36 h, followed by treatment with 1 μM Ang II for 24 h to mimic cell injury.

### Clinical sample collection

Plasma samples from patients with cardiac hypertrophy (n = 15) were obtained from the Affiliated Drum Tower Hospital. The study was approved by the Ethics Committee of The Affiliated Drum Tower Hospital. In addition, informed consent was provided by all patients.

### Bioinformatics analysis

The online tool CircInteractome (https://circinteractome.nia.nih.gov/index.html) was used to predict the target circRNAs [30]. In addition, TargetScan 7.2 (http://www.targetscan.org/vert_72/) and miRWalk (http://zmf.umm.uni-heidelberg.de/apps/zmf/mirwalk/micrornapredictedtarget.html) were used to predict the downstream target of miR-145-5p [31,32].

### Luciferase reporter assay

293 T cells (1.0x10^4^ cells/well) were inoculated in 96-well plates. For circNfix and miR-145-5p, wild-type or mutant circNfix fragments were inserted into the pmirGLO Dual-Luciferase miRNA Target Expression Vector (Promega, Madison, WI, USA). Cells were then transfected with circNfix-wild-type or circNfix mutant plasmids (600 ng), along with 20 nM miR-145-5p or control mimics. Similarly, for ATF3 and miR-145-5p, the wild-type or mutant ATF3 3ʹ untranslated region fragment was cloned into the pmirGLO Dual-Luciferase miRNA Target Expression Vector. Cells were transfected with ATF3-wild-type or ATF3 mutant plasmids (500 ng), along with 20 nM control or miR-145-5p mimics. A dual-luciferase system (Promega, Madison, WI, USA) was used to assess the luciferase activity after 48 h of transfection [33]. miR-145-5p mimics, control mimics, miR-145-5p inhibitor and control inhibitor were provided by Shanghai GenePharma Co., Ltd.

### *RNA fluorescence* in situ *hybridization (FISH)*

Cardiomyocytes were fixed with paraformaldehyde (4%), followed by treatment with 0.5% Triton. FISH was performed using a FISH Tag™ RNA Multicolor Kit (Invitrogen, USA), according to the manufacturer’s instructions. 4ʹ,6-diamidino-2-phenylindole (DAPI) was used to stain the cell nucleus. The localization of circNfix was observed using a Leica SP5 Spectral scanning laser confocal microscope (Leica Microsystems, Wetzlar, Germany) [12].

### RNA pull-down assay

The biotinylated probe for miR-145-5p and control probe were synthesized by Hanbio Biotechnology Co., Ltd. The probe was used to coat the streptavidin-coated beads. Cardiomyocytes were then lysed and pretreated with magnetic beads; RNA and beads were mixed. Following washing with binding buffer three times, TRIzol® reagent (Invitrogen; Thermo Fisher Scientific, Inc.) was used to isolate the RNA, and the results were analyzed via reverse transcription-quantitative polymerase chain reaction (RT-qPCR), as described previously [12].

### RT-qPCR

TRIzol reagent was used to isolate the RNA from tissues or cells. Next, 1.0 μg RNA was reverse-transcribed into cDNA using a reverse transcription kit (Takara Bio, Inc.). RT-qPCR was then conducted using a PCR Master mix in a 7900HT PCR system (Thermo Fisher Scientific, Inc.). The relative levels were evaluated using the 2^−ΔΔCq^ method. U6 or GAPDH was used for normalization, as described previously [34]. The primer sequences used are listed in Table 1.

**Table 1.** The information of primers

Name		Primer sequences
U6	Forward	5ʹ-CTCGCTTCGGCAGCACAT-3’
	Reverse	5ʹ-AACGCTTCACGAATTTGCGT-3’
miR-145-5p	Forward	5ʹ-GCATCTCTGGTCAGTTGGG-3ʹ
	Reverse	5ʹ-GACCTCAAGAACAGTAT-3ʹ
GAPDH	Forward	5ʹ-CACAGATCTGATGGATTTCAAGA-3’
	Reverse	5ʹ-TGCTGTCACCTTCACCGTTC-3’
circNfix	Forward	5ʹ-AGGAGATGCGGACATCAAAC-3ʹ
	Reverse	5ʹ-GTGAAATACGGGCTCGACTG-3ʹ
ATF3	Forward	5ʹ-TTTGCTAACCTGACGCCCTT-3ʹ
	Reverse	5ʹ-TGACTGATTCCAGCGCAGAG-3ʹ

### Western blot analysis

Total protein was isolated and quantified using a Nanodrop 8000 system (Thermo Fisher Scientific, Inc.). Sodium dodecyl sulfate-polyacrylamide gel electrophoresis (SDS-PAGE) gel (10%) was prepared and 40 μg protein was loaded for electrophoresis; the protein was then transferred onto a polyvinylidene fluoride (PVDF) membrane. Next, the blots were blocked in 5% skimmed milk at room temperature for 2 h and incubated with the primary antibody (1:1,000; Biosciences) overnight, followed by incubation with the secondary antibody (1:2,000; OriGene Technologies, Inc.) for a further 2 h. Finally, the blots were visualized using an electrochemiluminescence (ECL) kit (Cytiva), as described previously [12]. The data were analyzed using Image-Pro Plus 6.0 software (Media Cybernetics, Inc., Rockville, MD, USA).

### Immunofluorescence

Briefly, cardiomyocytes were fixed in 4% paraformaldehyde and permeabilized with 0.5% Triton X-100 for 30 min. They were then incubated with α-smooth muscle actin (α-SMA) primary antibody overnight at 4°C, followed by incubation with fluorochrome-conjugated secondary antibodies for 2 h at room temperature [34]. DAPI staining was performed for 5 min to identify the cell nucleus. Next, the stained cells were observed under a microscope (Olympus CX23 Tokyo, Japan).

### Histological and terminal deoxynucleotidyl transferase-mediated dUTP nick end labeling (TUNEL) analysis

The mice were sacrificed with CO_2_, and the heart tissues of the mice were collected. The tissues were fixed with 4% paraformaldehyde, embedded and sectioned. The size and morphological alterations in the heart were evaluated by H&E staining, as described previously [29]. TUNEL analysis was conducted using a Cell Death Detection kit (Roche Diagnostics Corporation, Indianapolis, IN, USA), according to the manufacturer’s instructions. Finally, the findings were observed under a microscope (Leica DM4 B, Shanghai, China).

### Echocardiography in mice

Echocardiography in mice was performed 4 weeks post-TAC using the Vevo 2100 Imaging system, as described previously [29]. Briefly, mice were treated with 50 mg/kg sodium pentobarbital (1%) and placed on an examination board with the chest facing up; all the hair was shaved. The 7.5 MHz probe was used; the left ventricular (LV) dimensions were measured with M-mode using the scan head and from more than three cardiac cycles. Left ventricular inner diameters during diastole and systole were determined from the M-modes and heart rate. Diastolic and systolic volumes were obtained by applying Simpson’s rule of discs to the serially acquired short-axis images. Stroke volume was calculated using the following method: Stroke volume = diastolic volume – systolic volume. Ejection fraction was calculated using the following formula: Ejection fraction = (stroke volume/diastolic volume) x 100%. The cardiac output was determined using the following equation: Cardiac output = stroke volume x heart rate.

### Statistical analysis

The experimental data were analyzed using GraphPad Prism software (GraphPad Software, Inc.). The results are presented as the mean ± standard deviation, and each assay was repeated at least three times. Comparisons between two or more groups were conducted using Student’s t-test or one-way analysis of variance (ANOVA), followed by Tukey’s post-hoc test [12]. P < 0.05 was considered to indicate a statistically significant difference.

## Results

### Characterization of circNfix in cardiomyocytes

**Figure 1.**
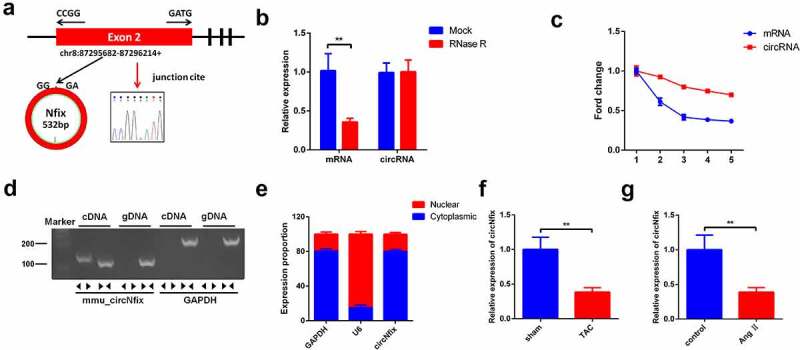
**Characterization of circNfix in cardiomyocytes**. (a) CircNfix was back-spliced by exons 2 of Nfix gene as the black arrow showed. The red arrow indicates head-to-tail splicing junction site of circNfix. (b) The expressions of linear mRNA Nfix and circNfix in RNase R-treated cardiomyocytes were detected with RT-qPCR. (c) RT-qPCR analysis of circNfix and Nfix mRNA in actinomycin D-treated cardiomyocytes. (d) CircNfix has been validated using nucleic acid electrophoresis. (e) The distribution of circNfix in cardiomyocytes was examined by RT-qPCR. (f, g) RT-qPCR was used to detect the expression of circNfix in TAC-treated mice and in Ang II treated cardiomyocytes. **P < 0.01, compared with control group; n = 3

CircRNAs have been found to play a critical role in cardiac hypertrophy [29,35]. However, the association between circNfix and cardiac hypertrophy remain largely unknown. Therefore, the aim of the present study was to explore the role of circNfix in cardiac hypertrophy. First, the profile of circNfix was characterized. As indicated in Figure 1(a), circNfix (532 bp; circBase ID: hsa_circ_0005660) was derived from exon 2 of the Nfix gene. Next, the stability of circNfix was evaluated by treating circNfix and Nfix mRNA with RNase R (an exoribonuclease that digests linear RNAs but not circRNAs). The results suggested that the amount of linear Nfix was markedly decreased following RNase R treatment. However, circNfix was resistant to RNase R, indicating that circNfix was indeed circular (Figure 1(b)). In addition, actinomycin D was used to prevent transcription and evaluate the half-life of the linear Nfix and circNfix. The results indicated that circNfix was much more stable than the linear form (Figure 1(c)). Furthermore, nucleic acid electrophoresis illustrated that circNfix could only be amplified by cDNA but not gDNA priming (Figure 1(d)).

Next, RNAs from the cytoplasm and nucleus were separated; the location of circNfix in cardiomyocytes was investigated using RT-qPCR. The results demonstrated that circNfix was mainly located in the cytoplasm (Figure 1(e)). Of note, it was found that the expression of circNfix in TAC-treated mice and Ang II-treated cardiomyocytes was significantly downregulated, compared with their respective control groups (Figure 1(f,g)). Collectively, these data suggested that circNfix was an abundant, stable and circular transcript in cardiomyocytes.

### *Overexpression of circNfix protects cardiomyocytes against Ang II* in vitro

**Figure 2.**
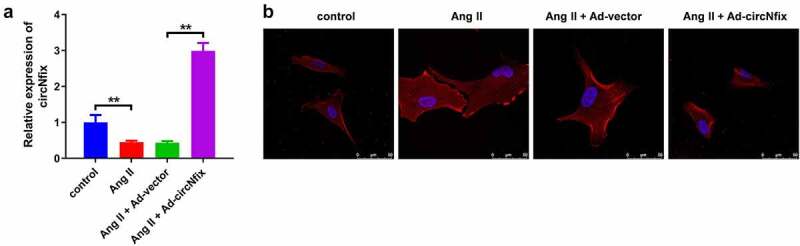
**CircNfix protected cardiomyocytes against Ang II *in vitro***. (a) Transduction efficiency of adenovirus *in vitro* has been examined by RT-qPCR. (b) The immunofluorence staining was used to analyze the size of cardiomyocytes in different group. **P < 0.01; n = 3

**Figure 3.**
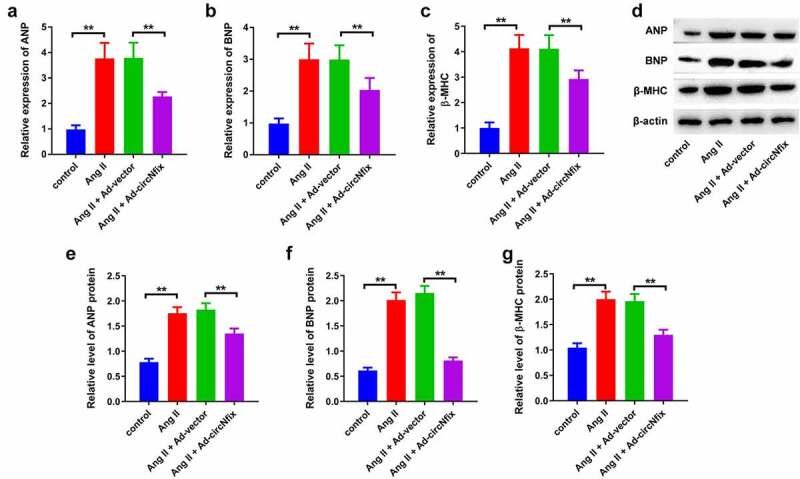
**CircNfix protected cardiomyocytes against Ang II *in vitro* via downregulation of ANP, BNP and β-MHC. (A, B and C)** The expressions of cardiac hypertrophic markers including ANP, BNP and β-MHC were evaluated by RT-qPCR. **(D, E, F and G)** The expressions of cardiac hypertrophic markers including ANP, BNP and β-MHC were detected with Western blotting. **P < 0.01; n = 3

In order to explore the role of circNfix in cardiac hypertrophy, cardiomyocytes were treated with Ang II to mimic the pathogenesis of the disease. The efficiency of the adenovirus was first evaluated using RT-qPCR. As shown in Figure 2(a), circNfix expression was significantly elevated in cardiomyocytes following ad-circNfix transduction, indicating the high transduction efficiency of ad-circNfix *in vitro*. In addition, Ang II treatment markedly induced hypertrophy in cardiomyocytes, an effect that was completely reversed by ad-circNfix treatment (Figure 2(b)). Moreover, Ang II promoted the expression of hypertrophic markers (atrial natriuretic peptide (ANP), B-type natriuretic peptide (BNP) and β-myosin heavy chain (β-MHC)) in cells [36]; however, these increases were all inhibited by ad-circNfix (Figure 3(a-g)). Collectively, ad-circNfix could protect cardiomyocytes against Ang II *in vitro*.

### Overexpression of circNfix protects mice against TAC-induced cardiac hypertrophy

**Figure 4.**
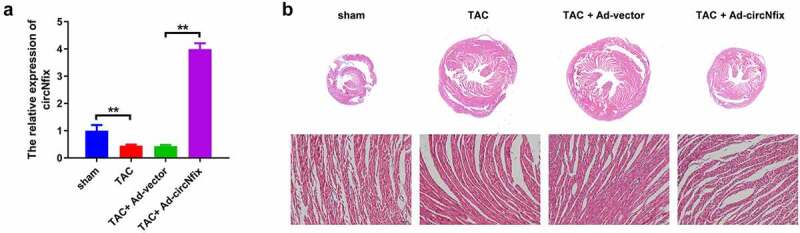
**CircNfix protected mice against TAC-induced cardiac hypertrophy**. (a) The expression of circNfix in myocardium was analyzed by RT-qPCR. (b) HE staining was performed to evaluate the size of the heart and cardiomyocytes. **P < 0.01; n = 3

**Figure 5.**
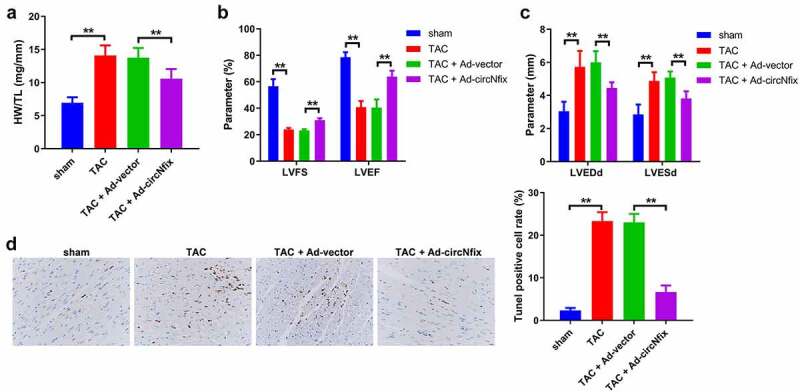
**CircNfix protected mice against TAC-induced cardiac hypertrophy via alleviation of cardiac function**. (a) Quantification of HW/TL was calculated. (b, c) Examination of the echocardiographic parameters was performed. (d) Cell apoptosis was calculated using TUNEL assay. **P < 0.01; n = 3

The function of circNfix was next investigated in TAC-induced cardiac hypertrophy *in vivo*. The expression of circNfix in mouse cardiac tissue was evaluated using RT-qPCR. As indicated by RT-qPCR, TAC decreased the expression level of circNfix, while ad-circNfix markedly promoted circNfix expression in the myocardium (Figure 4(a)). In addition, TAC-induced cardiac hypertrophy was significantly reversed by ad-circNfix (Figure 4(b)). Furthermore, TAC increased the HW/TL ratio, which was reversed by ad-circNfix (Figure 5(a)).

Next, an ultrasound system was used to measure echocardiographic parameters of the mice, including left ventricular fraction shortening (LVFS), left ventricular ejection fraction (LVEF), left ventricular end-diastolic diameter (LVEDd) and left ventricular end-systolic diameter (LVESd). The results indicated that TAC treatment markedly impaired the cardiac function of the mice; however, TAC-induced cardiac dysfunction was alleviated by ad-circNfix (Figure 5(b,c)). Consistently, TAC-induced apoptosis of cardiomyocytes was decreased by ad-circNfix (Figure 5(d)). In combination, these results showed that ad-circNfix could protect mice from TAC-induced cardiac hypertrophy.

### circNfix sponges miR-145-5p in 293 T cells

**Figure 6.**
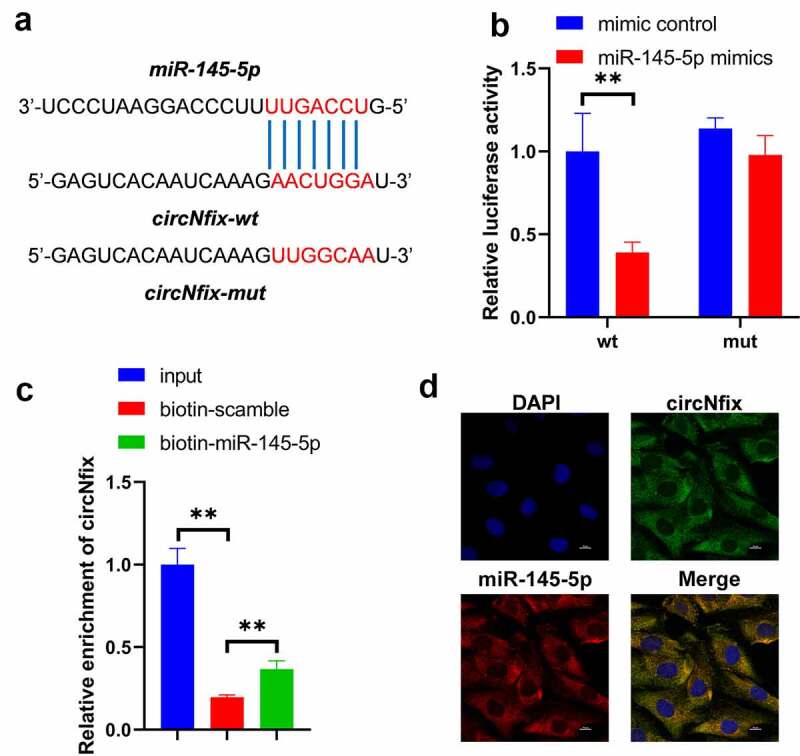
**CircNfix sponged miR-145-5p in cardiomyocytes**. (a) The predicted binding sequence between circNfix and miR-145-5p. (b) Luciferase assay was performed to detect the interaction between circNfix and miR-145-5p. (c) RNA pull down was performed to detect the interaction between circNfix and miR-145-5p. (d) FISH assay with specific probes targeting miR-145-5p and circNfix has been conducted to detect the co-location of miR-145-5p and circNfix in cardiomyocytes. **P < 0.01; n = 3

To explore the mechanism via which ad-circNfix protected mice against TAC-induced cardiac hypertrophy, bioinformatics tools were used. The results showed that circNfix/miR-145-5p duplex might exhibit the minimum free energy, indicating that miR-145-5p may be the target of circNfix (Figure 6(a)). In addition, the luciferase reporter assay results showed that miR-145-5p mimics markedly decreased the luciferase activity in cells transfected with the wild-type sequence of circNfix; however, miR-145-5p mimics had no effect on luciferase activity in cells transfected with the mutant sequence of circNfix (Figure 6(b)). Meanwhile, RNA pull-down assay results suggested that the miR-145-5p probe could enrich the expression of circNfix compared with the control probe, indicating the direct interaction between circNfix and miR-145-5p (Figure 6(c)). In addition, FISH assay revealed that circNfix was co-localized with miR-145-5p in the cytoplasm (Figure 6(d)). These data indicated that circNfix sponged miR-145-5p in cardiomyocytes.

### miR-145-5p mimics can reverse the protective effects of circNfix in Ang II-treated cardiomyocytes

**Figure 7.**
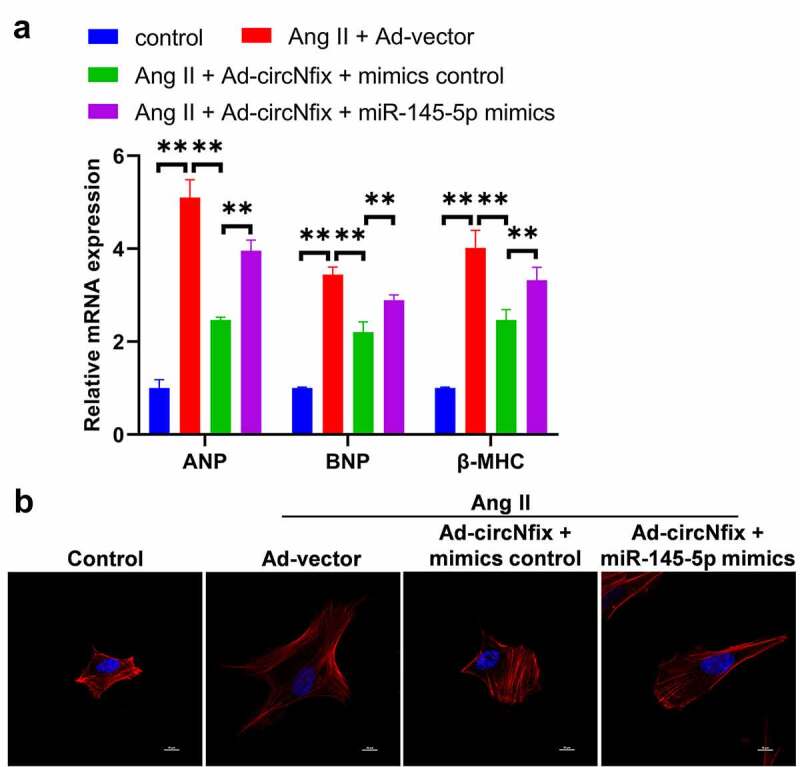
**MiR-145-5p mimics could reverse the protective effects of circNfix in Ang II-treated cardiomyocytes**. (a) The expressions of cardiac hypertrophic markers including ANP, BNP and β-MHC were evaluated by RT-qPCR. (b) The immunofluorence staining was used to analyze the size of cardiomyocytes in different group. **P < 0.01; n = 3

To confirm the interaction between circNfix and miR-145-5p, miR-145-5p mimics were used for plasmid transfection. As shown in Figure 5(a), miR-145-5p mimics notably reversed the effects of circNfix on the expression of BNP, ANP and β-MHC in Ang II-treated cardiomyocytes (Figure 7(a)). Consistently, miR-145-5p mimics significantly inhibited the anti-hypertrophy effect of circNifx in Ang II-treated cardiomyocytes (Figure 7(b)). These findings suggested that miR-145-5p mimics could reverse the protective effects of circNfix Ang II-treated cardiomyocytes.

### miR-145-5p targets ATF3 in cardiomyocytes

**Figure 8.**
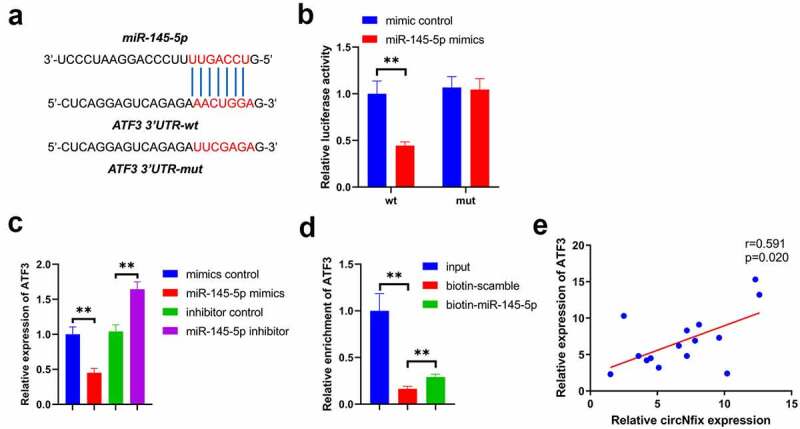
**ATF3 was a target of miR-145-5p**. (a) The predicted binding sequence between miR-145-5p and ATF3 was presented. (b) Luciferase assay was performed to detect the interaction between miR-145-5p and ATF3. (c) RNA pull down was performed to detect the interaction between miR-145-5p and ATF3. (d) Pearson’s correlation analysis was performed to detect the correlation between ATF3 and circNfix. **P < 0.01; n = 3

Next, the potential targets of miR-145-5p were explored using online bioinformatics tools, and ATF3 was predicted to be the target of miR-145-5p (Figure 8(a)). In addition, the luciferase reporter assay results confirmed the direct targeting relationship between miR-145-5p and ATF3 (Figure 8(b)). Next, the effect of miR-145-5p mimics on the expression of ATF3 in cardiomyocytes was examined with RT-qPCR. As indicated in Figure 8(c), miR-145-5p mimics significantly decreased the expression of ATF3 in cardiomyocytes; by contrast, miR-145-5p inhibitor increased the level of ATF3. Furthermore, the RNA pull-down assay results confirmed that miR-145-5p directly bound with ATF3 (Figure 8(d)). Pearson correlation coefficient revealed a positive correlation between circNfix and ATF3 in clinical samples (Figure 8(e,f)). In combination, these data indicated that miR-145-5p targeted ATF3 in cardiomyocytes.

## Discussion

Evidence has shown that circRNAs play an important role in cardiovascular diseases, including cardiac hypertrophy [35,37]. Garikipati et al found that circFndc3b could alleviate cardiac repair after myocardial infarction through targeting FUS/VEGF-A axis [38]. Wu et al indicated that downregulation of circRNA TLK1 attenuated neuronal injury after brain ischemia via sponging miR-335-3p [39]. Meanwhile, xu et al found that downregulation of circHIPK3 could attenuate pressure overload-triggered cardiac hypertrophy via targeting miR-185-3p [29]. Cardiac hypertrophy is a pathophysiological process, which is defined as an increase in the myocardial mass [40,41]. However, the functions of circRNAs in cardiac hypertrophy remain largely unknown. In this study, we identified a circRNA originating from exon 2 of its host gene Nfix, and found that circNfix was significantly downregulated in TAC-treated mice and Ang II-treated cardiomyocytes. In addition, overexpression of circNfix could suppress pressure overload-induced cardiac hypertrophy *in vitro* and *in vivo*.

Evidences have shown that, circNfix plays a critical role in various human diseases [28,42]. Ding et al found that circNfix could promote the progression of glioma *in vitro* and *in vivo* via sponging miR-378e [42]. Lu et al showed that circNfix could promote the viability, migration and invasion of lung cancer cells [43]. Cheng et al indicated that downregulation of circNfix suppressed cell proliferation, invasion and migration in pituitary adenomas [44]. Meanwhile, a previous study indicated that circNfix silencing promoted cardiac regeneration in the mice subjected to myocardial infarction [28]. However, in the present study, it was demonstrated that circNfix attenuated pressure overload-induced cardiac hypertrophy. The discrepancy suggested that circNfix might play a different role when the heart is subjected to different stresses.

It has been shown that cardiac hypertrophy leads to an upregulation in the expression of several hypertrophic biomarkers including β-MHC, ANP and BNP [45]. Liu et al found that Ang II could induce cardiomyocyte hypertrophy via upregulation the levels of ANP, BNP, β-MHC; the present results were consistent with these findings [46]. In addition, we found that Ang II significantly upregulated the expressions of ANP, BNP, β-MHC in cardiomyocytes; however, these effects were reversed by circNfix overexpression. Consistently, Xu et al found that Ang II increased the levels of ANP, BNP, β-MHC in cardiomyocytes, while these changes were reversed by circHIPK3 knockdown [29]. Meanwhile, Chen et al showed that downregulation of lncRNA DSCAM-AS1 could decrease the levels of ANP, BNP, β-MHC in Ang-II-treated cardiomyocytes [45]. All these data indicated that circNfix could protect cardiomyocytes against Ang II *in vitro* via downregulation of ANP, BNP, β-MHC levels.

Significantly, mice with cardiac hypertrophy displayed higher levels of LVEDd and LVESd, and lower levels of LVFS and LVEF [2]. Evidences have shown that mice subjected to TAC characterized by the decreased LVFS and LVEF and the increased LVEDd and LVESd [47,48]. In this study, we found that TAC could impair the cardiac function of the mice via elevation of LVEDd and LVESd and reduction of LVFS and LVEF; however, TAC-induced cardiac dysfunction was alleviated by circNfix overexpression, which were consistent with the previous reports [29]. These data showed that overexpression of circNfix could protect mice against TAC-induced cardiac hypertrophy *in vivo* via downregulation of LVEDd and LVESd and elevation of LVFS and LVEF.

It has been shown that circRNAs could exert various biological functions via acting as miRNA sponges to regulate downstream genes expression [49,50]. In this study, we found that miR-145-5p might be sponged by circNfix, which was verified by Luciferase reporter assay and RNA pull-down assay. In addition, we found that circNfix protected mice against TAC-induced cardiac hypertrophy by sponging miR-145-5p. Of note, ATF3 was confirmed as a direct target of miR-145-5p. In conclusion, circNfix attenuated pressure overload-induced cardiac hypertrophy by regulating the miR-145-5p/ATF3 axis.

ATF3 is a vital member of the cAMP response element-binding protein/ATF family [51–53]. ATF3 exerted regulatory functions at the transcriptional level either as a promoter or an inhibitor [53]. Recent studies have reported that ATF3 is activated during heart failure [54,55]. Consistent with the results of those studies, it was found herein that circNfix and ATF3 were positively correlated in patients with cardiac hypertrophy, indicating the key role of ATF3 in the progression of cardiac hypertrophy. In addition, it was first confirmed that ATF3 was targeted by miR-145-5p, which acts as a competing endogenous RNA of circNfix. These findings extended our understanding of the effect of ATF3 in cardiac hypertrophy and the pathogenesis of the disease. However, this study was not without its limitations. First, the interaction between ATF3 and cardiac hypertrophy remains unclear, and additional rescue experiments should be performed. Secondly, the clinical significance of circNfix, miR-145-5p and ATF3 during the development of cardiac hypertrophy should be explored in the future. Thirdly, Yang et al found that testosterone could inhibit the proliferation in Ang II-treated cardiac fibroblasts via regulation of ERK1/2 signaling pathway [56]. Shi et al found that inhibition of PTEN could promote cardiac hypertrophy [57]. Thus, further study is needed to investigate whether circNfix could regulate the progression of cardiac hypertrophy via targeting other genes, such as ERK, PTEN.

## Conclusion

In the present study, circNfix was found to attenuate pressure overload-induced cardiac hypertrophy by regulating the miR-145-5p/ATF3 axis. The present findings enhanced our understanding of the biological effect of circNfix, miR-145-5p and ATF3 in the progression of cardiac hypertrophy.

## Data Availability

All the data are available from the corresponding author due to reasonable request.

## Highlights

1. CircNfix was downregulated in cardiac hypertrophy.
2. CircNfix could attenuate pressure overload-induced cardiac hypertrophy.
3. CircNfix/miR-145-5p/ATF3 axis regulates cardiac hypertrophy progression.
4. CircNfix might serve as a potential target for prevention of cardiac hypertrophy.
